# Comparison between the Efficacy of Sacral Erector Spina Plane Block and Pudendal Block on Catheter-Related Bladder Discomfort: A Prospective Randomized Study

**DOI:** 10.3390/jcm13123617

**Published:** 2024-06-20

**Authors:** Bilge Olgun Keleş, Elvan Tekir Yılmaz, Ali Altınbaş

**Affiliations:** Anesthesiology and Reanimation Department, Giresun University Faculty of Medicine, 28100 Giresun, Turkey; elvanty28@hotmail.com (E.T.Y.); ali.altinbas@hotmail.com (A.A.)

**Keywords:** catheter-related bladder discomfort, peripheral nerve block, pudendal nerve block, regional anesthesia, ultrasound-guided sacral erector spinae plane block, transurethral resections prostate

## Abstract

**Objective:** Catheter-related bladder discomfort (CRBD) due to indwelling urinary catheterization in patients undergoing transurethral resection of the prostate (TURP) is difficult to tolerate and needs to be treated. This randomized prospective study aimed to compare the efficacy of sacral erector spinae plane block (SESPB) and pudendal nerve block (PNB) in reducing the incidence and score of CRBD. **Methods:** This study was conducted between November and December 2023. ASA I-III, fifty-four TURP patients were divided into two groups: Group 1 received SESPB (n = 27) and Group 2 received PNB (n = 27) under ultrasound guidance at the end of surgery. The incidence of CRBD, CRBD score, numerical rating scale (NRS) score, use of rescue analgesics, block performance time, first call for analgesics, patient satisfaction, and side effects were recorded for 24 h. **Results:** The incidence of CRBD was lowest at 33.3% and highest at 48.1% in Group 1 and lowest at 25.9% and highest at 48.1% in Group 2, with no significant difference between the groups at all measurement times. CRBD scores and NRS scores were low and similar between the two groups. Block performance times were 9 ± 1.7 min in SESPB and 20 ± 2.5 min in PNB, and there was a significant difference between the mean times (*p* < 0.001). Patient satisfaction was adequate and similar in both groups. **Conclusions:** SESPB demonstrated a similar decreasing effect to PNB on the incidence and scores of CRBD in the first 24 h following TURP operations. The duration of SESPB administration was shorter than PNB.

## 1. Introduction

Transurethral resection of the prostate (TURP) is the most common surgical treatment for benign prostatic hypertrophy [1]. After TURP, a 20 or 22 Fr foley urethral catheter is inserted and left in place for at least 24 h. The most common complication of indwelling catheterization is catheter-related bladder discomfort (CRBD), with incidence rates ranging from 47 to 90%. CRBD is defined as urinary urgency, suprapubic pain, and burning sensation. It significantly reduces postoperative quality of life and patient satisfaction [2]. Predictors of CRBD include age over 50 years, male gender, history of indwelling catheter, lack of postoperative analgesics, catheter over 18 Fr, and lack of lubrication during insertion [3].

In the mechanism of CRBD, the catheter causes an increase in parasympathetic nerve activity in the bladder and increases acetylcholine release. Increased aceytlcholine stimulates muscarinic receptors (M2,3) in the bladder smooth muscle and causes involuntary contractions. The catheter in the continuously contracting bladder causes irritation of the bladder wall, and thus, CRBD occurs [2,3,4]. Many drugs and methods have been studied to reduce CRBD and studies are ongoing [5]. Muscarinic antagonists such as tolterodine have been studied, and efficacy has been achieved, and dry mouth, facial flushing and blurred vision have been encountered as side effects [6]. Studies with ketamine, an anesthetic, were successful, but hallucinations were frequent [7]. Tramadol, which has a muscarinic receptor inhibition feature among analgesics, and gabapentin and pregabalin among antiepileptics have been found to reduce CRBD, but sedation and postoperative nausea and vomiting (PONV) side effects were found to be higher in all of them when compared with control groups [8,9]. Intraoperative paracetamol is also one of the analgesics that has been shown to reduce CRBD [10]. Neuraxial blocks such as epidural tramadol and intrathecal morphine have been used with success, but the incidence of unwanted side effects such as motor block, hypotension, epidural hematoma, and respiratory depression could not be ignored [11,12]. TAP block, dorsal penile block, and pudendal block were used as peripheral nerve blocks to reduce CRBD. The highest success and longest duration of effect were achieved with pudendal block [12,13,14,15,16].

The pudendal nerve consists of the ventral branches of sacral nerve 2–4. It innervates the perineum. The dorsal penile nerve, the terminal branch of the pudendal nerve, is known to innervate the membranous urethra [17]. Anatomically, afferent nerves to the urethra and bladder triangle originate from the sacral somatic nerves S2–4, and theoretically, these nerves should be blocked to reduce CRBD. Studies have been performed using ultrasound-guided pudendal nerve block to reduce CRBD in TURP surgery, and its use has shown success [14,15,16]. Furthermore, studies have shown that pudendal block is effective for postoperative analgesia in genitourinary surgery [18,19]. 

Sacral ESPB (SESPB) was first described in 2019 by Tulgar [20] using a bilateral method and provided postoperative analgesia in pilonidal sinus surgery. Later, Aksu and Gürkan [21] described the midline SESPB method and used it for analgesia in urogenital surgeries. Both methods have shown analgesic efficacy in different cases in the urogenital and anorectal regions innervated by the pudendal nerve [22,23].

Based on clinical studies in the literature, we hypothesized that the pudendal nerve arising from S2–4 can be blocked by SESPB and be as effective as PNB in reducing the incidence and scores of CRBD after TURP.

This study aimed to compare the efficacy of SESPB and PNB on the incidence and scores of CRBD.

## 2. Materials and Methods

### 2.1. Patients and Methods

This prospective randomized study was completed between November–December 2023 at Giresun Training and Research Hospital. Approval was obtained from the Giresun Training and Research Hospital Clinical Research Ethics Committee on 5 June 2023 with the number 2023/7, and this study was conducted following the Declaration of Helsinki. The study protocol was registered in the clinical trials database (ClinicalTrials gov ID: NCT06127394). Informed written consent was obtained from the patients before surgery. This study followed CONSORT guidelines. 

Fifty-four patients of the American Society of Anesthesiologists (ASA) physical status I-III group, aged 18 years and above, and undergoing elective TURP were included in this study. Patients who were excluded from participating in this study had a history of previous prostate or bladder surgery, urinary tract infection, neurogenic or hyperactive bladder; were users of anticholinergic drug and opioid analgesics; had known local anesthetic allergies; or had an infection or anatomical changes in the lumbosacral region and perineum. 

### 2.2. Randomization and Blindness

Randomization was performed using a closed envelope technique based on computer-generated random numbers. Each group consisted of 27 patients and was named Group 1: Ultrasound (US)-guided midline SESPB and Group 2: Neurostimulator (NS)- and US-guided bilateral PNB. An experienced anesthesiologist opened the envelope administered the block according to the group code. The patients were blinded to which block was administered to them, and postoperative follow-up was performed by an anesthesiologist blinded to the groups.

### 2.3. Anesthetic Management

All patients were premedicated with intravenous midazolam 0.5 mg/kg before surgery. Peripheral oxygen saturation (SpO2) measurements, electrocardiograms, and non-invasive blood pressure monitoring were performed in the operating room. Anesthesia induction was performed with 1 mcg/kg fentanyl and 2 mg/kg propofol, and a laryngeal mask of an appropriate size was placed according to the patient’s weight and mouth structure. Anesthesia was maintained with 2% sevoflurane, 50% air/oxygen mixture, and 0.1–1 mcg/kg/min remifentanil infusion. At the end of the operation, an 18–20 fr, 3-lumen bladder catheter was inserted, and continuous bladder irrigation was started with 0.9% saline. All patients were given 1000 mg paracetamol and 1 mg/kg tramadol iv intraoperatively and 15 mg/kg paracetamol at intervals of 8 h postoperatively as a multimodal analgesia plan. Ondansetron was administered at 4 mg to prevent PONV. After the blocks were applied to the groups, anesthesia was terminated, and the patients were awakened and transferred to the recovery room. 

### 2.4. US-Guided Midline ESPB

**Figure 1:**
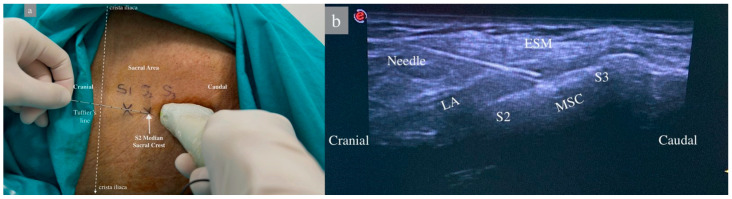
Patient position and sonoanatomy during ultrasound-guided sacral erector spinae plane block. (**a**) Patient in lateral decubitis position. The dashed white lines represent Truffier’s line, a transverse line connecting the upper parts of the crista iliacae. S1–2–3: Level of 1st, 2nd, and 3rd sacral vertebrae median crests. (**b**) ESM: Erector Spinae Muscles, LA: Local Anesthetic, MSC: Median Sacral Crest, S2–S3: Level of sacral vertebrae.

In group 1, the patient was placed in the lateral decubitus position, and the sacral region was sterilized with povidone-iodine and draped. A sheath was applied to the linear US probe, and the probe was positioned longitudinally in the midline at the level of the spinous process of the 5th lumbar vertebra. The median sacral crest was observed, and the 1st and 2nd median sacral crest and erector spinae muscles were visualized by moving the probe caudally. A 22G, 80 mm (Stimuplex Ultra360 B. Braun, Melsungen, Germany) was advanced in a craniocaudal direction using an in-plane technique until the tip of the needle touched the second sacral crest as described by Aksu [15]. After negative aspiration, 2 mL of saline was administered, the needle position was confirmed by the US, and 30 mL of 0.25% bupivacaine was administered between the sacral crest and the erector spinae muscle, and muscle elevation was observed (Figure 1).

### 2.5. NS- and US-Guided Anterior Approach Bilateral PNB

**Figure 2:**
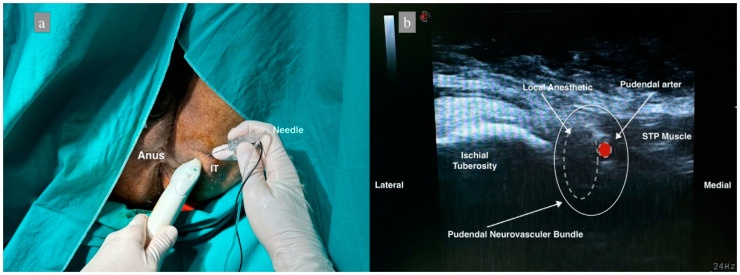
Patient position and sonoanatomy during ultrasound-guided pudendal nerve block. (**a**) Patient in lithotomy position, IT: Ischial Tuberosity. (**b**) Dashed white lines represent the distribution of local anesthetic, dashed red figure shows the pudendal artery, STP: Superficial transverse perineal muscle.

In group 2, the perianal area was sterilized and closed at the end of surgery with the patient in the lithotomy position. The linear ultrasound probe was sheathed. The ischial bulge was palpated, and the ultrasound probe was placed obliquely. The probe was moved medially, and the sacrotuberous ligament was observed. After identifying the pudendal artery below the sacrotuberous ligament by Doppler, a 22 G 80 mm needle (Stimuplex Ultra360 B.Braun, Melsungen, Germany) was inserted using the in-plane technique and advanced 4–5 cm. NS was used to avoid intraneuronal injection, and the current intensity was determined as 0.5 mA since it was performed under US guidance. If any twitch was obtained, the needle was withdrawn 1 mm. Following negative aspiration, the needle position was confirmed with 2 mL of saline, and the block was performed with 10 mL of 0.25% bupivacaine. The same procedure was performed on the contralateral side (Figure 2).

### 2.6. Follow-Up and Outcomes

The primary outcomes were the incidence and severity of postoperative CRBD in patients. CRBD was assessed at 30 min and 1, 2, 4, 8, 12, 12, and 24 h using a scoring system developed and used by Agarwall et al. [6] “0: No symptoms, 1: Mild, tolerable discomfort, 2: Moderate, discomfort present but verbalized, 3: Severe, discomfort intolerable and accompanied by body movements”. CRBD below 2 was considered low, 2 and above was considered high, and 1 mg/kg tramadol IV was planned as rescue analgesia. The incidence of CRBD was determined as none and present (mild, moderate, severe). For this symptom, which is reported to occur between 47–90% in the literature, less than 47% was considered low.

Secondary outcomes were block performance time, NRS scores, rescue analgesic use, and first call for analgesics. 

Block performance time included a period starting with skin cleansing and ending with the administration of all local anesthetics to the patient. Unlike in group 1, the time needed to move patients from the lithotomy position to the lateral decubitus position was added to the total time. In group 2, repositioning was not necessary because the patient was already in the lithotomy position.

The NRS scores used to evaluate postoperative pain were evaluated 30 min and 1, 2, 4, 8, 12, and 24 h after surgery on a scale of “0: no pain” to “10: the most severe pain experienced”. The difference between NRS and CRBD was explained to the patients before surgery to avoid confusion. If NRS ≥ 4, 1 mg iv morphine was planned as the other rescue analgesic.

Intraoperative and postoperative data (heart rate (HR), mean arterial pressure (MAP), SpO2 values) were recorded every 10 min until discharge from the recovery unit. Demographic data (ASA, gender, age), duration of surgery, side effects (PONV, allergic reaction, hypotension), complications (vascular-nerve injury, hematoma, local anesthetic systemic toxicity), and patient satisfaction were recorded. Patient satisfaction was assessed 24 h postoperatively using a questionnaire (0: not at all satisfied, 10: very satisfied).

### 2.7. Sample Size

Data from a similar study by Wang SY et al. [16] investigating the difference in the incidence of CRBD between transholium laser therapy pudendal block and control groups were used to determine the sample size. The minimum number of patients to be included in this study was calculated as 24 in each group using G*Power software (latest ver. 3.1.9.7; Heinrich-Heine-Universität Düsseldorf, Düsseldorf, Germany) with z-test at 90% power and 0.05% type error level. Considering the possibility of exclusion of 10–15% of the patients, a total of 54 patients, 27 in each group, were decided to be included in this study (Table 1).

### 2.8. Statistics

IBM SPSS V23 was used for statistical analysis. A Shapiro–Wilk test was used to examine the normality of quantitative data. An independent sample *t*-test was used to compare normally distributed data, and a Mann–Whitney U test was used to compare non-normally distributed data. Qualitative data were compared using the Pearson chi-square test. The data are presented as mean ± standard deviation, median (minimum–maximum), and n (%). Statistical significance was accepted as *p* < 0.05.

## 3. Results

**Figure 3:**
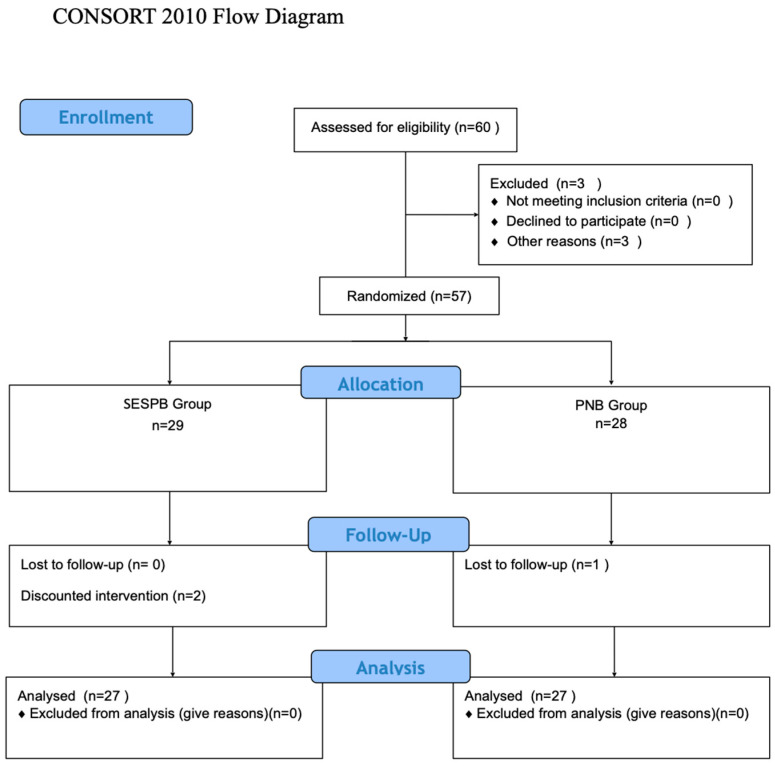
Consort flow diagram.

This study included 60 patients (Figure 1). A total of 3 patients were excluded [operation was postponed due to hypertension (n = 3)], and 57 patients were randomized. In the SESPB group, one patient’s operation was changed, one patient was excluded because the block could not be performed, and one patient was lost to follow-up in the PNB group. The study was completed with a statistical analysis of the remaining 54 patients, with 27 in each group (Figure 3).

Both intraoperative and postoperative data (HR, MAP, and SpO2) and demographic characteristics were similar between the two groups. The duration of the surgery was also similar. Block performance time was 9 ± 1.7 min in the SESPB group and 20 ± 2.5 min in the PNB group, and there was a significant difference between the mean times (*p* < 0.001). The number of patients using tramadol as rescue analgesic was seven (25.9%) in Group 1 and nine (33.3%) in Group 2. Morphine, a second rescue analgesic, was not used in any patient. The first call for analgesic was 7.5 ± 2.6 h in Group 1 and 8.6 ± 4.3 h in Group 2. Patient satisfaction was similar between the groups. PONV was observed in three patients in group 1 and allergic reaction in one patient in group 2 (Table 2). No complications related to the procedures were observed.

The incidence of CRBD was lowest at 33.3% and highest at 48.1% in Group 1 and lowest at 25.9% and highest at 48.1% in Group 2. There was no significant difference between the groups in terms of CRBD incidence and scores at all measurement times (*p* > 0.05). NRS scores were low in both groups at all measurement times, and there was no difference between the groups (Table 3). 

## 4. Discussion

This is the first study comparing SESPB and PNB in terms of CRBD incidence and scores and NRS scores after TUR-P. The incidence and severity of CRBD and NRS scores decreased in both blocks, and no significant difference was observed between the groups. The block performance time of SESPB was found to be significantly shorter than PNB.

Li Xiaoqiang et al. [15] performed PNB to reduce CRBD in TURP cases and compared it with the control group and found that the incidence of CRBD was 52% to 64% in the pudendal group and 69% to 90% in the control group and was statistically significant at all times. Wang SY [16] found that VAS scores, incidence, and severity of CRBD decreased with PNB use in Holep surgery. The incidence was found to be 3.6–36.4% in the PNB group and 38.2–76.4% in the control group.

Similarly, Göğer YE et al. [14] applied PNB to reduce CRBD after TURP and showed that both VAS and CRBD scores decreased. Many other studies have been conducted on this subject, and PNB was found to be successful compared to the control group in almost all of them [18,19]. In our study, the lowest incidence of CRBD was found to be 25.9% and the highest was 48.1% in the PNB group, which is consistent with the literature. SESPB was similar to PNB, and the lowest incidence was 33.3% and the highest was 48.1%. 

The highest incidence of CRBD in our patients was at the 8th hour, which is consistent with the 6–8 h duration of action of nerve blocks in general. 

For pain palliation in genitourinary surgery, either the pudendal nerve or the ventral branches of the S2–4 nerves should be blocked. In genitourinary surgeries, SESPB has been applied by the midline approach and single injection, and successful results have been obtained [20,21,22,23]. In a cadaveric study, we have demonstrated that the radiopaque substance administered with the midline SESPB method passes to the anterior region of the sacrum and the involvement of the S2 to S5 nerves with Computed Tomography [24]. We also utilized the findings of our cadaveric study in planning this study.

Kukreja et al. blocked the pudendal nerve using midline SESPB in a sex reassignment operation with very difficult pain management and said that no nerve block other than neuraxial blocks alone can show this efficacy in both the perineum and genital area. They stated the reasons for not preferring PNB as the risk of nerve damage, bilaterality, and clinicians’ unwillingness to perform PNB. Therefore, they showed SESPB as a good alternative to pudendal block [22]. 

In a study comparing SESPB, penile block and caudal block for hypospadias surgery, SESPB was more effective than penile block and provided analgesia equivalent to caudal block, but caudal block had more side effects [25]. When PNB was previously performed with traditional methods, the risk of injury to organs such as the vessels, bowels, and bladder was high. US guidance of the block has significantly reduced this risk. The risk of local anesthetic toxicity was also reduced by monitoring the distribution of local anesthetics [26]. The use of US guidance with NS also reduces the complication of nerve injury by preventing intraneural injection [27]. We did not encounter any complication such as vascular-organ-nerve injury by performing our block under US guidance with NS, which is the safest method of PNB.

Compared with other ESPBs, SESPB has no vascular network, pleura, or nearby organs, so the risk of complications is lower. In addition, all ESP blocks can be safely performed in coagulopathic patients and patients on anticoagulants due to the simple technique and ultrasound guidance [28]. We did not encounter any complications such as hematoma, local anesthetic toxicity, or intramuscular injection in our SESPB patients.

In our study, a statistically significant difference was found between block performance times in favor of SESPB. Even if the time to change position was added to the total time in the SESPB group, its shorter duration was considered as an advantage. While it was easier to obtain images with US in the lateral decubitus position, it took time to determine the injection site in the lithotomy position for PNB. We think that single injection in SESPB and bilateral injection in PNB also prolonged the time. Prolonged block performance time is undesirable because it may result in both prolonged anesthesia time and surgeons’ reluctance to perform the block because of time loss.

### Limitations

An important limitation of our study is the absence of a control group in which no block was performed because ethical approval could not be obtained. 

Due to the position of the patients, performing the block while awake was considered undesirable for the patient and not preferred by clinicians, so PNB was performed while the patients were asleep. Therefore, block success and dermatome spread could not be measured.

## 5. Conclusions

In the study findings, SESPB was found to have similar efficacy to PNB on the incidence and scores of CRBD in the first 24 h following TURP operations. SESPB was considered to be an effective choice in TURP due to its decreasing effect on CRBD incidence and scores, effective postoperative analgesia, shorter administration time, easy administration technique with a single injection, and low complication risk.

## Figures and Tables

**Table 1:** Power analysis data.

We Performed the Power Analysis	A Priori
On the primary outcome of	Catheter-related bladder discomfort incidence at 30 min and 1, 2, 4, 8, 12, and 24 h after the surgery
Based on the two-tailed statistical test	z-test, proportions
And accepting the cutoff for significance (α)	0.05
And a power (1-β) of	0.9
The variability of the primary outcome was:	0.45
Based on data taken from	A previous randomized controlled study ^1^
We considered as clinically relevant a difference of	0.45
Consequently, the effect size was	0.90
The total sample size needed was	48 (%10–15 dropouts, 54 patients planned)

^1^ A study by Wang SY et al. [16].

**Table 2:** Data on demographic and clinical characteristics of patients according to groups.

Variables	Group SESPB (n = 27)	Group PNB (n = 27)	*p* Value
ASA ps (median (min-max))	2 (2–2)	2 (2–2)	1
Age, years (mean ± SD)	67.2 ± 12.1	70.5 ± 7.0	0.391
Weight, kg (mean ± SD)	73.4 ± 9.4	74.9 ± 12.0	0.664
Duration of surgery, minutes (mean ± SD)	86.11 ± 10.77	84.4 ± 14.56	0.720
Block performing time, minutes (mean ± SD)	9 ± 1.7	20 ± 2.5	<0.001 *
Rescue analgesic tramadol use n (%)	7 (25.9)	9 (33.3)	0.782
First call for analgesics time, hours (mean ± SD)	7.5 ± 2.6	8.6 ± 4.3	0.525
Patient with side effects n (%)	3 (11.1)	1 (3.7)	0.299
Patient satisfaction (median (min–max))	9 (6–10)	9 (8–10)	0.327

* *p* < 0.001. ASA ps: American Society of Anesthesiologist physical status. SESPB: Sacral Erector Spinae Plane Block. PNB: Pudendal Nerve Block.

**Table 3:** Comparison of incidence and severity of CRBD and NRS scores between groups.

Time	30 min		1 h		2 h		4 h		8 h		12 h		24 h	
Group	1	2	1	2	1	2	1	2	1	2	1	2	1	2
Total	27	27	27	27	27	27	27	27	27	27	27	27	27	27
None	17 (63.0)	18 (66.7)	16 (59.3)	16 (59.3)	16 (59.3)	16 (59.3)	17 (63.0)	19 (44.4)	14 (51.9)	14 (51.9)	14 (51.9)	13 (48.1)	18 (66.7)	20 (74.0)
CRBD	10 (37.0)	9 (33.3)	11 (40.7)	11 (40.7)	11 (40.7)	11 (40.7)	10 (37.0)	8 (29.6)	13 (48.1)	13 (48.1)	12 (44.1)	13 (48.1)	9 (33.3)	7 (25.9)
Mild	10 (37.0)	9 (33.3)	9 (33.3)	10 (37.0)	9 (33,3)	10 (37)	8 (29.6)	7 (25.9)	8 (29.6)	9 (33.3)	8 (29.6)	10 (37)	8 (29.6)	7 (25.9)
Moderate	0 (0)	0 (0)	2 (7.4)	1 (3.7)	2 (7.4)	1 (3.7)	2 (7.4)	1 (3.7)	4 (14.8)	4 (14.8)	4 (14.8)	3 (11.1)	1 (3.7)	0 (0)
Severe	0 (0)	0 (0)	0 (0)	0 (0)	0 (0)	0 (0)	0 (0)	0 (0)	1 (3.7)	0 (0)	0 (0)	0 (0)	0 (0)	0 (0)
*P*	0.776		0.333		0.733		0.467		0.647		0.538		0.867	
NRS	0 (0–3)	0 (0–2)	0 (0–3)	0 (0–2)	0 (0–3)	1 (0–3)	0 (0–2)	1 (0–3)	0 (0–3)	1 (0–3)	0 (0–3)	0 (0–2)	0 (0–2)	0 (0–2)
*P*	0.882		0.773		0.173		0.454		0.562		0.938		0.398	

CRBD incidence and severity data are expressed as the number of patients n (%), NRS data are expressed median (min-max). CRBD: Catheter-Related Bladder Discomfort, NRS: Numeric Rating Scale, Group 1: Sacral Erector Spinae Plane Block, Group 2: Pudendal Nerve Block.

## Data Availability

All data presented in this article are included in the manuscripts or tables/figures. Further inquiries can be directed to the corresponding author.
